# Deciphering Necroptosis-Associated Molecular Subtypes in Acute Ischemic Stroke Through Bioinformatics and Machine Learning Analysis

**DOI:** 10.1007/s12031-024-02241-3

**Published:** 2025-01-02

**Authors:** Zongkai Wu, Hongzhen Fan, Lu Qin, Xiaoli Niu, Bao Chu, Kaihua Zhang, Yaran Gao, Hebo Wang

**Affiliations:** 1 Department of Neurology, Hebei General Hospital, Shijiazhuang, China; 2 Hebei Provincial Key Laboratory of Cerebral Networks and Cognitive Disorders, Hebei General Hospital, Shijiazhuang, China; 3 Department of Neurology, Hebei Medical University, Shijiazhuang, China

**Keywords:** Acute ischemic stroke, The necroptosis-related differentially expressed genes, Functional enrichment analyses, Weighted gene co-expression network analysis, The Least absolute shrinkage and selection operator regression model, Competitive endogenous RNA

## Abstract

Acute ischemic stroke (AIS) is a severe disorder characterized by complex pathophysiological processes, which can lead to disability and death. This study aimed to determine necroptosis-associated genes in acute ischemic stroke (AIS) and to investigate their potential as diagnostic and therapeutic targets for AIS. Expression profiling data were acquired from the Gene Expression Omnibus database, and necroptosis-associated genes were retrieved from GeneCards. The differentially expressed genes (DEGs) and necroptosis-related genes were intersected to obtain the necroptosis-related DEGs (NRDEGs) in AIS. In AIS, a total of 76 genes associated with necroptosis (referred to as NRDEGs) were identified. Enrichment analysis of these genes revealed that they were primarily enriched in pathways known to induce necroptosis. Using weighted gene co-expression network analysis (WGCNA), five co-expression modules consisting of NRDEGs were identified, along with two modules that exhibited a strong correlation with AIS. Protein–protein interaction (PPI) analysis resulted in the identification of 20 hub genes. The Least absolute shrinkage and selection operator (LASSO) regression model demonstrated promising potential for diagnostic prediction. The receiver operating characteristic (ROC) curve validated the diagnostic model and selected nine characteristic genes that exhibited statistically significant differences (*p* < 0.05). By employing consensus clustering, distinct patterns of necroptosis were identified using these nine signature genes. The results were validated by quantitative PCR (qPCR) in venous blood from patients with AIS and healthy controls and HT22 cells, as well as external datasets. Furthermore, the analyzed ceRNA network included nine lncRNAs, six miRNAs, and three mRNAs. Overall, this study offers novel insights into the molecular mechanisms underlying NRDEGs in AIS. The findings provide valuable evidence and contribute to our understanding of the disease.

**Supplementary Information:**

The online version contains supplementary material available at 10.1007/s12031-024-02241-3.

## Introduction

Stroke is a significant global health issue, leading to disability in adults and causing a substantial number of deaths worldwide (Johnson et al. 2019). It poses a considerable burden at both individual and societal levels. An episode of cerebral ischemia arises when a clot or thrombus obstructs the blood supply to the brain, leading to an acute ischemic stroke (AIS). During thrombolytic therapy for AIS, ischemia/reperfusion (I/R) injury is likely to occur. I/R injury triggers oxidative stress and inflammation responses, leading to further neuronal damage (Nagy and Nardai 2017). Currently, AIS can be effectively managed with intravenous thrombolysis and mechanical thrombectomy to restore blood flow in occluded vessels. However, the prognosis heavily relies on the timely administration of treatment. Despite the effectiveness of current treatment options in preventing long-term disability in AIS patients, accurate prognostic prediction remains challenging. Existing models for prognostic prediction in AIS patients often lack reliable predictive capabilities (Fahey et al. 2018; Quinn et al. 2017). The diagnosis of AIS primarily relies on neuroimaging techniques due to the absence of efficient, rapid, and accurate diagnostic biomarkers. Early diagnosis and successful treatment are crucial for reducing mortality rates and improving outcomes in AIS. Therefore, there is an urgent need for systematic studies to understand the biological processes involved in AIS.

Cell death plays a crucial role in regulating infarction during ischemia and I/R injury. Neurons in ischemic stroke undergo rapid necrotic cell death (Xing et al. 2012). Therefore, our understanding of the mechanisms underlying neuronal death in ischemic stroke is vital as it can provide valuable insights into potential targets that can be controlled. Necroptosis is a recently identified pathway of programmed cell death that can be induced by the activation of inflammatory receptors such as tumor necrosis factor receptor 1, toll-like receptor (TLR), and Fas/CD95 (Vercammen et al. 1998a, 1998b; Holler et al. 2000; Matsumura et al. 2000; Liao et al. 2020). This process relies on the activation of receptor-interacting protein kinase (RIPK)1 through its interaction with RIPK3, resulting in the phosphorylation of the pseudokinase mixed lineage kinase domain-like protein (MLKL) by RIPK3 (Galluzzi et al. 2014; de Almagro and Vucic 2015). In the context of ischemia and I/R injury, neurons experience rapid depletion of adenosine triphosphate (ATP) due to the sudden lack of oxygen and glucose caused by ischemia. This depletion results in plasma membrane depolarization, which triggers the secretion of glutamate and subsequent induction of N-methyl-d-aspartate (NDMA) receptors (Vacher et al. 2008). The excitotoxicity caused by glutamate and lactic acidosis further activates RIPK1 (Liao et al. 2020; Zhan et al. 2019). When caspase-8 is inhibited, RIPK1 is recruited via its receptor-interacting protein (RIP) homotypic interaction motif (RHIM) domain and phosphorylates RIPK3, forming complex IIb (Grootjans et al. 2017). Ultimately, MLKL is phosphorylated and polymerized, leading to the induction of necroptosis (Grootjans et al. 2017). Previous research has shown that intracerebral injection of necrostatin-1 (Nec-1), a specific inhibitor of RIPK1, can block ischemic stroke-induced neuronal necroptosis and attenuate delayed ischemic brain injury in mice (Degterev et al. 2005). Additionally, various investigations using the oxygen–glucose deprivation/re-oxygenation (OGD/R) model have observed elevated levels of necroptosis markers, such as RIPK1, RIPK3, and MLKL, in neuronal cells (Chen et al. 2018b; Yang et al. 2017; Vieira et al. 2014; Li et al. 2020b; Tang et al. 2018). It has been shown that the use of microarrays has described distinct changes in gene expression in whole blood from 0 to 24 h after ischemic stroke. (Tang et al. 2006; Stamova et al. 2014). Over time, many of the changes in interleukins have been directly quantified in the peripheral blood of patients with IS (Nayak et al. 2012; Perini et al. 2001). Macrophage migration inhibitory factor (MIF) derived from peripheral blood is induced to upregulate and promote endothelial cell apoptosis and necroptosis through RIPK1 kinase-dependent pathway after ischemic brain injury (Li et al. 2023). We elaborated on the links between peripheral blood gene expression and different pathological processes of stroke and ensured that the literature review reflected the latest advances in the field. Discussions of specific genes or signaling pathways were closely related to the goals and results of our analyses, reinforcing their significance as potential biomarkers. At the same time, we explained the rationale for the selection of peripheral blood samples and the relevance of this choice for understanding the biologic basis of CNS events. However, effective drugs targeting necroptosis for potential neuroprotection against ischemic brain injury are still lacking.

**Fig. 1:**
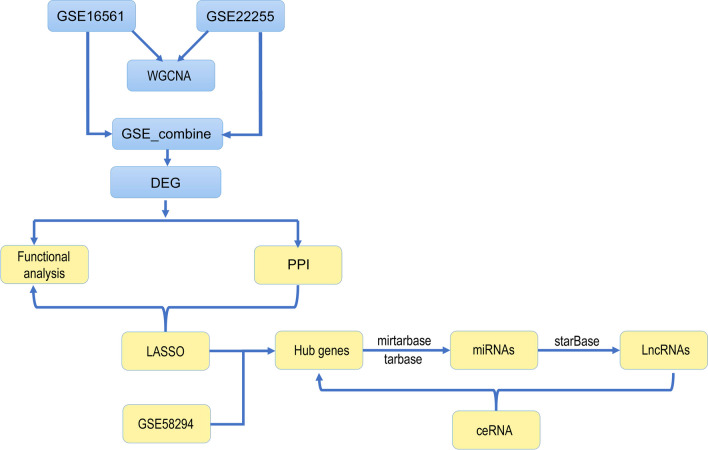
Schematic diagram of the research design

In this study, necroptosis-related differentially expressed genes (NRDEGs) in AIS were identified using a comprehensive analysis. Gene Ontology (GO), Kyoto Encyclopedia of Genes and Genomes (KEGG), Gene Set Enrichment Analysis (GSEA), and Weighted Gene Co-expression Network Analysis (WGCNA) were used to identify the molecular mechanism(s) of the NRDEGs in AIS. Next, a protein–protein interaction (PPI) network as well as the efficient and diagnostic Least absolute shrinkage and selection operator (LASSO) regression models were constructed. The effectiveness of the diagnostic model was confirmed by analyzing receiver operating characteristic (ROC) curves. A total of nine potential NRDEGs were identified as significant indicators for predicting the occurrence of AIS. To further investigate the immune component, we conducted an immune infiltration analysis and examined the correlations between the expression of these nine DEGs and 22 immune cell types. Additionally, we selected three DEGs to establish a competing endogenous RNA (ceRNA) network. Altogether, our study successfully unraveled the molecular mechanisms of necroptosis in AIS and established a foundation for its diagnosis and treatment. For a visual representation of our study design, please refer to Fig. 1 in the article.

## Materials and Methods

### Identification of DEGs and NRDEGs in AIS

The gene expression profiling dataset of peripheral whole blood specimens (GSE16561) was retrieved from the Gene Expression Omnibus (GEO) (Barrett et al. 2007) database, and divided into 39 ischemic stroke patients (Stroke) and 24 healthy control subjects (Control). Another GEO dataset of peripheral blood specimens (GSE22255) (Krug et al. 2012) was obtained and divided into 20 ischemic stroke patients and 20 normal patients. The two datasets were merged and batch effects were mitigated using the combat (package sva) function in R (Leek et al. 2012) and verified by principal component analysis (PCA). The third GEO dataset of peripheral blood specimens of Homo sapiens from GPL570 (GSE58294) (Stamova et al. 2014) was downloaded and divided into 69 ischemic stroke patients (group name: Stroke) and 23 normal patients (group name: Normal). As the validation dataset, it was used to validate the hub genes and the comparison map between the two groups was drawn.

To assess the differential impact of gene expression values on AIS, group comparisons were conducted using the R package limma (Ritchie et al. 2015). For differential expression analysis, the following additional thresholds were used: logFC > 0.3 and adjP-value < 0.05 for upregulated genes (up_regulated_genes) and logFC <  − 0.3 and adjP-value < 0.05 for downregulated genes (down_regulated_genes).

We identified necroptosis-associated genes using GeneCards (Stelzer et al. 2016), which is an integrative and comprehensive database of human genetic information. A search using the term “necroptosis” yielded 630 necroptosis-related genes (Table S1).

A Venn diagram was drawn using the DEGs and necroptosis-related genes and the intersecting genes were identified as NRDEGs in AIS. These genes were then subjected to PPI network analysis.

### DEG Function and Pathway Enrichment Analyses

GO (Ashburner et al. 2000), an international standard for gene functional classification, is widely used as a tool for functional annotation and enrichment analyses. GO terms are classified as cellular component (CC), molecular function (MF), and biological process (BP). KEGG database is comprised of the pathways of experimentally validated metabolic processes and gene sets of human diseases, and it stores an extensive collection of data on genomes, biological pathways, drugs, chemicals, and diseases. The DEGs underwent GO term and KEGG pathway analyses using the clusterProfiler (Yu et al. 2012) in R package. *p* < 0.05 was deemed statistically significant.

### GSEA Evaluation

GSEA, a widely recognized computational method, is frequently employed to assess variations in pathway activities and biological processes within expression datasets. It determines whether a predefined set of genes exhibits notable differences between two biological states (Subramanian et al. 2005). GSEA was conducted on the gene expression data in the combined dataset of GSE16561 and GSE22255 using the clusterProfiler R package in order to study the biological differences between patients with the AIS and Control groups. The “c2.all.v7.5.2.entrez.gmt” gene set was obtained from the Molecular signatures database (MSigDB) (Liberzon et al. 2015) for GSEA of the combined dataset of GSE16561 and GSE22255. Adjusted *p* < 0.05 was deemed statistically significant. Both p-value and normalized enrichment score (NES) values are presented.

### Establishment of Co-expression Modules of DEGs Through WGCNA

WGCNA (Langfelder and Horvath 2008) is a systematic biological approach used to characterize the patterns of inter-gene correlations across samples in order to discover modules of highly correlated genes. Therapeutic targets or candidate biomarker genes were identified according to the intensity of the gene set as well as the relationship between the gene set and the phenotype. We analyzed the GSE16561 and GSE22255 datasets using the WGCNA package in R software. For the GSE16561 dataset, we set the minimum gene number, cut height and optimal soft-thresholding power to 50, 135 and 5, respectively, merged modules at a cut height of 0.95, and established a minimum distance of 0.2. For the GSE22255 dataset, the minimum gene number, cut height and optimal soft-thresholding power were set to 50, 80, and 7, respectively; modules were merged at a cut height of 0.4, and the minimum distance was fixed at 0.2. Using this approach, we successfully obtained co-expression modules for the DEGs within the two groups of both datasets.

### Development of PPI Network

STRING (Szklarczyk et al. 2019) was used to search for known and predicted PPIs and was employed to construct the PPI networks of both DEGs and NRDEGs.

Cytoscape v3.6.1 (Smoot et al. 2011), a well-known open-source software platform that integrates interaction networks, was utilized for the visualization of PPI networks. Cytoscape CytoHumba plugin (Chin et al. 2014) was used to study the network’s hub genes and identified the top 10 genes in the maximum correlation coefficient (MCC). Functional correlations for the key genes were calculated using the R package GOSemSim (Yu 2020).

### Construction of Diagnostic Model via LASSO Regression Model

LASSO regression involves the simultaneous screening of variables and complexity adjustment to fit a generalized linear model. Through regularization, a shrinkage penalty is introduced to limit the coefficients. The regularization process uses the sum of the absolute values of all the feature weights, which improves the interpretability of the model to a certain extent. A LASSO regression model was built for the genes in the co-expression modules of the DEGs using the glmnet package in R software (Engebretsen and Bohlin 2019; Mazumder and Hastie 2012). During the model construction process, we carefully screened the selected features and identified the best model for building a diagnostic model for cerebral infarction. Subsequently, we determined that the genes included in the model were the distinctive genes associated with cerebral infarction. To validate the models, ROC curves were drawn using the pROC package (Robin et al. 2011). A box plot was generated to display the characteristic genes of peripheral blood samples from patients with cerebral infarction and normal control patients. The genes with *p* < 0.05 were selected for visualization.

### Molecular Subtype Analysis of Cerebral Infarction

Consensus clustering is an algorithm based on resampling techniques. Its purpose is to identify individual members and their respective subgroup numbers, while also assessing the reliability and validity of the clustering data. The previously identified NRDEGs based on the combined dataset of GSE16561 and GSE22255 were defined as key necroptosis-associated genes. The ConsensusClusterPlus package (Wilkerson and Hayes 2010) in R software was employed to determine the gene expression profiles of the previously screened NRDEGs, and the cluster with the best clustering was selected. Based on these results, different necroptosis patterns were identified.

### Immune Infiltration Analysis

The CIBERSORT package (Chen et al. 2018a) was employed, utilizing a deconvolution algorithm with linear support vector regression, to predict the expression matrix associated with immune cell subtypes. This approach was applied to evaluate immune cell infiltration in patients with ischemic stroke and the control group using RNA-seq data. The differential enrichment of immune cells between patients with AIS and the control group was identified in the combined dataset of GSE16561 and GSE22255. Furthermore, we determined the Pearson correlation coefficients between immune cells and nine NRDEGs and examined the relationship between NRDEGs and the levels of immune infiltration.

### Network Construction for the Interactions Among Hub-mRNA, Hub-microRNA (miRNA), and Hub-Long Non-coding RNA (lncRNA)

We conducted an analysis of lncRNA and miRNA expression, focusing on their interaction with hub genes at the post-transcriptional level. To identify miRNA-mRNA targeting relationships, we utilized the miRTarBase database, which contains experimentally validated miRNA-target interactions (MTIs) from over 8500 articles (Huang et al. 2020). The database has been augmented with the newly released CLIP-seq dataset, resulting in over 500,000 MTIs. Leveraging improved natural language processing (NLP) technology, we collected additional target relationship pairs along with their network functions and annotation information. For identification of miRNAs potentially binding to hub genes, we utilized the miRTarBase 2020 database (https://mirtarbase.cuhk.edu.cn/) (Huang et al. 2020). Additionally, the TarBase database (version 8), which compiles experimentally validated miRNA targets across multiple species, was used to predict miRNAs interacting with hub genes (Karagkouni et al. 2018). A list of miRNAs predicted by both databases was generated.

To search for miRNA targets, we employed the starBase database, which incorporates high-throughput sequencing datasets (CLIP-seq and Degradome-seq) and offers various visualization tools to explore microRNA targets (Cai et al. 2014). The database includes extensive data on miRNA-mRNA, miRNA-ncRNA, RNA-RNA, and RBP-RNA interactions. Using the starBase database, we predicted lncRNAs interacting with miRNAs. An interaction network comprising hub lncRNAs, hub miRNAs, and hub-mRNAs was constructed, and the Cytoscape software was employed to visualize the network as a Sankey diagram.

### HT22 Cell Culture and Treatment

The HT22 cells (Zhejiang Ruyao Biotechnology Co. Ltd., Zhejiang, China), which are an immortalized mouse hippocampal neuronal cell line, were cultured in Dulbecco’s modified Eagle’s medium (DMEM, Corning, NY, United States) containing 10% fetal bovine serum (FBS, BI, Israel) and 1% double antibodies (37 °C, 5% CO_2_ cell culture incubator). When the cells attached to the wall and reached 80 to 90% confluence, the culture medium were discarded, washed once with PBS, and digested with 3 ml of trypsin for 2 min. The process of digestion was terminated by adding medium immediately after the cells became round and detached. The cells were transferred to a centrifuge tube, centrifuged at 1000 rpm for 5 min, re-suspended in fresh culture medium, and distributed at a 1:2 ratio. The original cells were transferred into a new cell culture bottle for continued culture, with medium changed every 2 to 3 days.

We divided the cells into both a control group and a model group, seeded them into 96-well plates at a density of 5 × 10^4^, with 3 replicate wells for each group, and then cultured overnight. After 24 h, we removed the original culture medium in the model group, washed the cells twice with PBS, and then added 300 μl of glucose-free, serum-free DMEM medium to each well before being incubated in an anaerobic culture box for 4 h. After oxygen–glucose deprivation treatment, the cells in the model group were restored to complete DMEM medium, and returned to e cell incubator at 37 °C and 5% CO_2_ for 2 h. The cells in the normal control group were continuously cultured in complete medium and a cell incubator at 37 °C, 5% CO_2_.

### Clinical Data Collection

A total of 6 patients diagnosed with acute ischemic stroke (AIS) in the emergency department of Hebei General Hospital from April 2023 to April 2024 were selected as the research subjects. The mean age of the patients was 66.67 ± 5.22 years. Inclusion criteria were the following: the diagnostic criteria were in line with the guidelines for AIS diagnosis and treatment in China and the results of MRI and/or CT. Exclusion criteria were intracerebral hemorrhage; severe hepatic and renal insufficiency; severe cardiopulmonary and functional impairment; malignancy; and autoimmune diseases. At the same time, 6 healthy people who underwent health screening in the physical examination center of the hospital at the same time were randomly selected as the healthy control group. The average was 65.83 ± 5.24 years. All patients with AIS were scored using the National Institutes of Health Stroke Scale (NIHSS) to assess the severity of neurological impairment. There was no significant age difference between the two groups (*p* > 0.05). There were no significant differences in age, gender, and risk factors such as diabetes, hypertension, and hyperlipidemia between the AIS group and the control group (Table 1).

**Table 1:** The demographic data of AIS

Groups	AIS (*n* = 6)	Control (*n* = 6)
Age (year)	66.67 ± 5.22	65.83 ± 5.24
Male, *n* (%)	3 (50%)	3 (50%)
Hypertension, *n* (%)	3 (50%)	3 (50%)
Diabetes, *n* (%)	3 (50%)	2 (33.33%)
Total cholesterol (mmol/l)	4.61 ± 1.01	3.79 ± 1.38
Triglyceride (mmol/l)	1.93 ± 1.18	1.64 ± 0.82
NIHSS score		
(1–4) (5–15) (16–25)	1 3 2	

This study has been approved by the Ethics Committee of Hebei General Hospital, and the study notification has been signed with patients and their families. Informed consent, ethics approval number was [2024(102)]. Whole blood from all subjects was collected and loaded into a 2 × 5 ml EDTA anticoagulant tube.

### Quantitative Polymerase Chain Reaction (qPCR Analysis)

The mRNAs were extracted from HT22 cells and venous blood (5 ml) collected from healthy controls and hospitalized AIS patients within 24 h by adding Trizol reagent. The Fast Plus RT Master mix reverse transcription reagent kit (Supersmart® 6-min Heat-resistant first-strand cDNA Synthesis Kit) was used to synthesize the extracted mRNAs into cDNAs, called the reverse transcription, RT reaction. PCR was carried out using the SYBR Green qPCR Mix pre-mixed qPCR reagent kit (Superbrilliant® Third generation ZAPA SYBR Green qPCR premix) to amplify the cDNA in a two-step process and perform fluorescence quantification, with the reaction conditions as follows: 95 °C for 300 s; 95 °C for 10 s; and 60 °C/65 °C for 20 s, for 40 cycles. The final primer concentration was 0.4 μM, with GAPDH as the reference gene. The relative expression levels were calculated using the 2^^(−△△Ct)^ method, and the melting curve of the products were analyzed to ensure reaction specificity (see Supplemental Files for primer sequences).

### Statistical Analysis

All statistical tests were conducted with R v4.0.2 (https://www.r-project.org). For normally distributed continuous variables, the statistical significance was assessed using an independent Student’s *t*-test. Non-normally distributed continuous variables were compared using the Mann–Whitney *U* test and the Wilcoxon rank sum test. All *p*-values were two-sided, and *p* < 0.05 was deemed statistically significant.

## Results

### DEGs in Patients with AIS

**Fig. 2:**
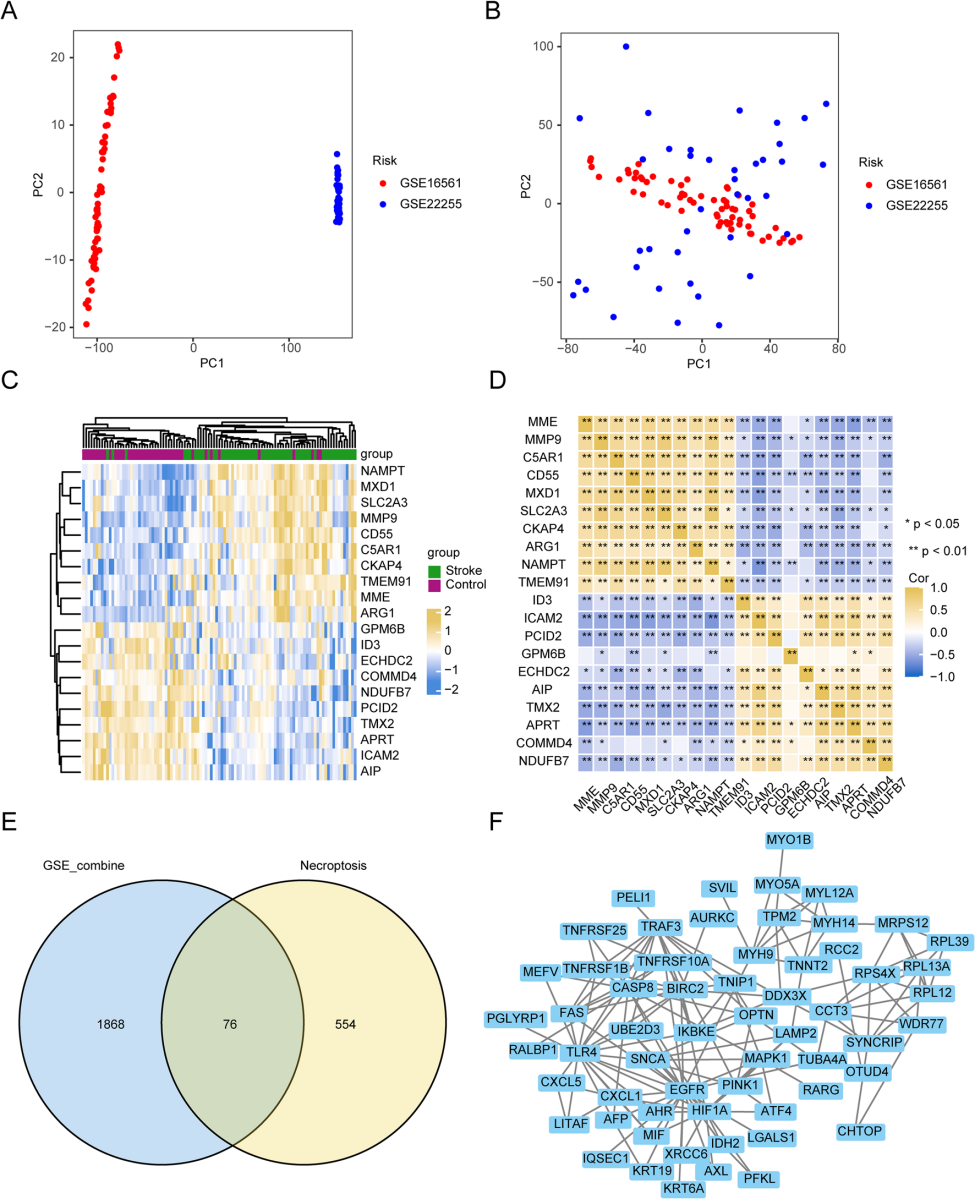
Differential expression analysis. **a** PCA analysis of GSE_combine prior to mitigating batch effects. **b** PCA analysis of GSE_combine after removing batch effects. **c** The abscissa and ordinate represent the patient ID and DEGs, respectively. Yellow, upregulated gene expression; blue, downregulated gene expression; green annotation bars, normal patients; dark red annotation bars, cerebral infarction patients. **d** Correlation of the DEGs. Yellow is positive correlation of genes; blue is negative correlation of genes. **e** The blue circle represents the DEGs of GSE_combine, and the yellow circle represents necroptosis-associated genes. The NRDEGs were identified by performing the intersection. **f** PPI network map of NRDEGs

To compare the patients with AIS with normal patients, we merged the two sets of GSE data as GSE_combine, and the batch effects were then eliminated. PCA analysis showed that batch effects had been removed from the datasets (Fig. 2a and b). For the identification of DEGs, differential expression analysis was carried out using the limma software package, with |logFC|> 0.3 and adjPvalue < 0.05. A total of 1945 DEGs were acquired, including 1195 upregulated and 750 downregulated genes. The ten up- and downregulated DEGs with the highest logFC are presented as heatmaps (Fig. 2c) and gene correlation coefficient heatmaps (Fig. 2d). We compared the DEGs obtained from the combined dataset of GSE16561 and GSE22255 and necroptosis-related genes to identify 76 NRDEGs (Table S2 and Fig. 2e). A PPI network of these 76 genes is shown in Fig. 2f.

### Functional and Pathway Enrichment Analyses

**Fig. 3:**
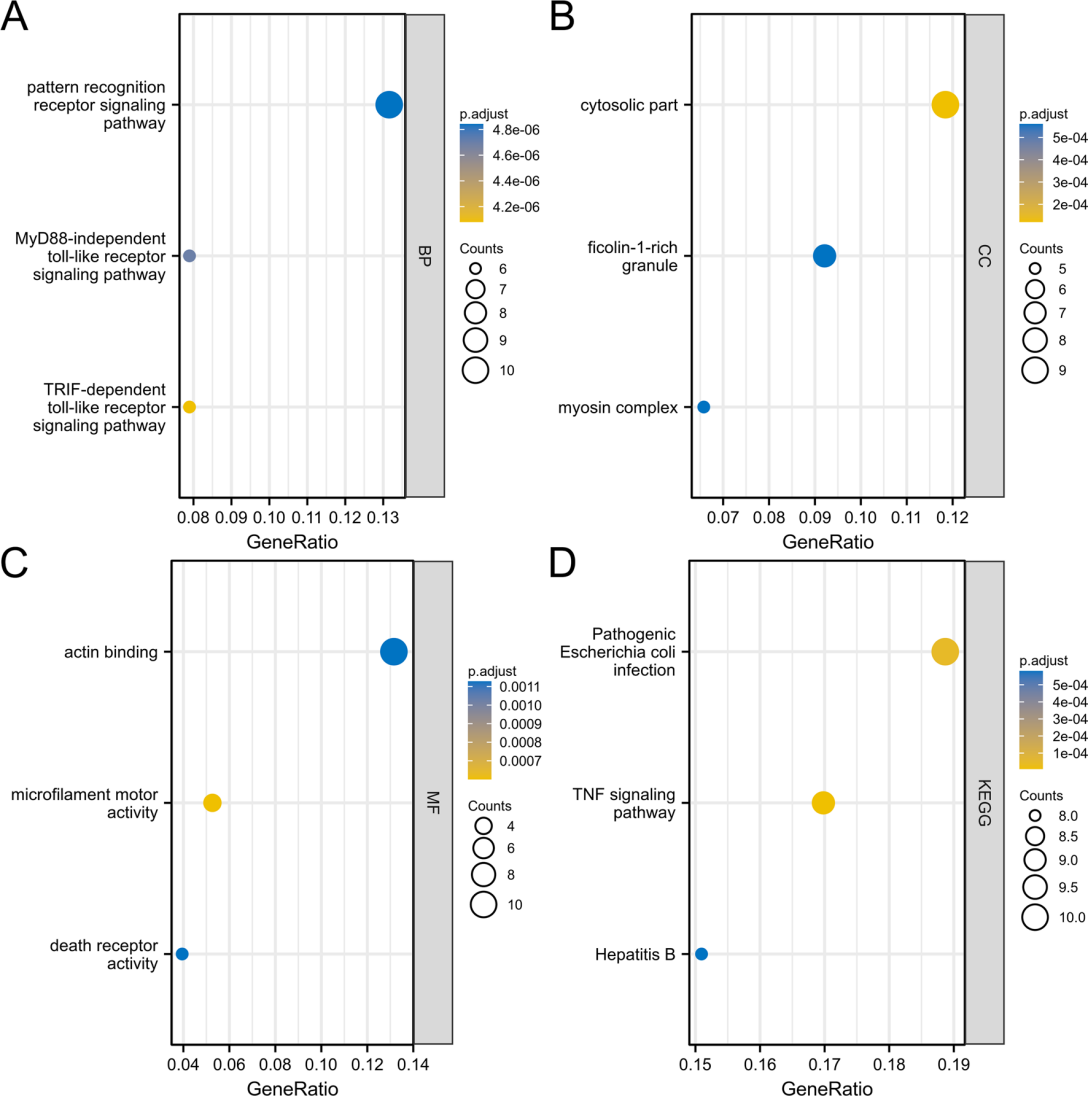
Functional and pathway enrichment analyses. **a**–**c** GO analysis. The ordinate is -log(p.adjust), the abscissa is the GO terms, and the node’s color indicates the p.adjust value. Less than 0.02, more blue; more than 0.01, more yellow. **d** KEGG analysis. The abscissa and ordinate represent the gene ratio and KEGG pathways, respectively. The node’s size corresponds to the number of genes in the enrichment pathway, and the node’s color indicates the p.adjust value. Less than 0.02, more blue; more than 0.01, more yellow

Next, GO functional enrichment analysis was conducted for the NRDEGs (Table 2). The DEGs related to AIS were mainly enriched in BP terms such as toll/interferon response factor (TRIF)-dependent TLR signaling pathway, myeloid differentiation factor 88 (MyD88)-independent TLR signaling pathway, and pattern recognition receptor signaling pathway (Fig. 3a); in CC terms such as cytosolic part, myosin complex, and ficolin-1-rich granule (Fig. 3b); and in MF terms such as microfilament motor activity, death receptor activity, and actin binding (Fig. 3c). Next, KEGG analysis demonstrated that the DEGs were enriched in biological pathways, such as hepatitis B, pathogenic *Escherichia coli* infection, and tumor necrosis factor (TNF) signaling pathway (Fig. 3d, Table 3).

**Table 2:** GO functional enrichment analysis of DEGs

Ontology	ID	Description	GeneRatio	BgRatio	pvalue	p.adjust	qvalue
BP	GO:0035666	TRIF-dependent toll-like receptor signaling pathway	6/76	29/18670	1.64e-09	4.08e-06	2.96e-06
BP	GO:0002756	MyD88-independent toll-like receptor signaling pathway	6/76	33/18670	3.78e-09	4.70e-06	3.41e-06
BP	GO:0002221	Pattern recognition receptor signaling pathway	10/76	197/18670	7.10e-09	4.84e-06	3.51e-06
CC	GO:0044445	Cytosolic part	9/76	247/19717	4.51e-07	1.21e-04	8.35e-05
CC	GO:0016459	Myosin complex	5/76	65/19717	5.13e-06	5.60e-04	3.87e-04
CC	GO:0101002	Ficolin-1-rich granule	7/76	185/19717	7.24e-06	5.60e-04	3.87e-04
MF	GO:0000146	Microfilament motor activity	4/76	22/17697	2.17e-06	6.00e-04	4.74e-04
MF	GO:0005035	Death receptor activity	3/76	11/17697	1.23e-05	0.001	8.91e-04
MF	GO:0003779	Actin binding	10/76	431/17697	1.50e-05	0.001	8.91e-04

**Table 3:** KEGG functional enrichment analysis of DEGs

Ontology	ID	Description	GeneRatio	BgRatio	pvalue	p.adjust	qvalue
KEGG	hsa04668	TNF signaling pathway	9/53	112/8076	3.65e-08	7.01e-06	4.96e-06
KEGG	hsa05130	Pathogenic *Escherichia coli* infection	10/53	197/8076	4.65e-07	4.46e-05	3.15e-05
KEGG	hsa05161	Hepatitis B	8/53	162/8076	9.07e-06	5.81e-04	4.11e-04

#### GSEA

**Fig. 4:**
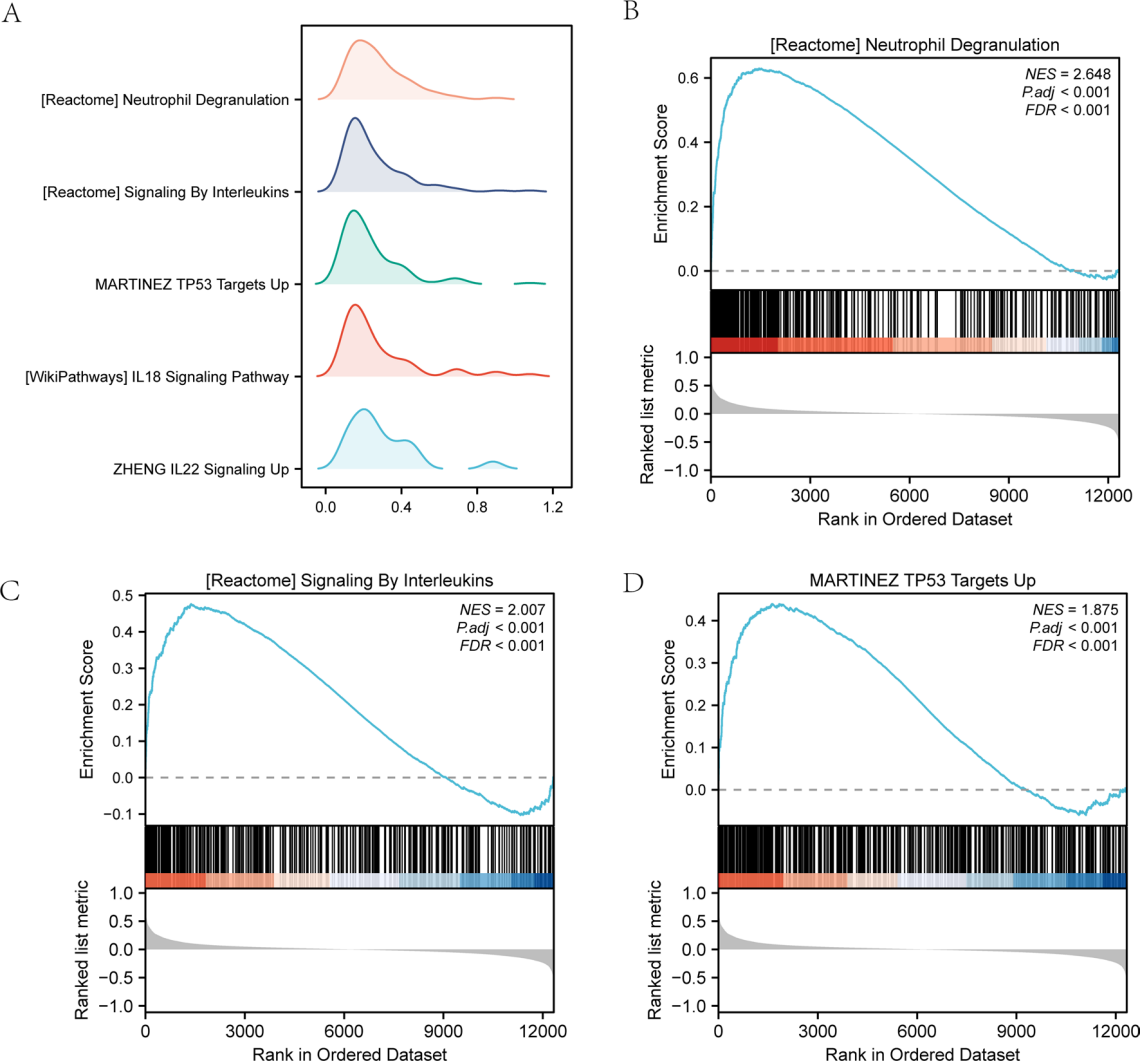
GSEA results. **a** GSEA analysis of the combined dataset of GSE16561 and GSE22255. The horizontal and vertical axes represent the gene ratio and the number of genes for each enriched GO terms, respectively. **b**–**d** GSEA showed enrichment in **b** neutrophil degranulation, **c** signaling by interleukins, and **d** G alpha (i) signaling

Through GSEA of the combined dataset, we explored the relationships between AIS and the DEGs’ BP, CC, and MF. The results of GSEA with adjPvalue < 0.05 showed an enrichment in neutrophil degranulation, G protein-coupled receptors (GPCR) ligand binding, signaling by interleukins, G alpha (i) signaling events, neuronal system, rhodopsin-like receptors (class A/1), and NABA-secreted factors (Fig. 4a–d, Table 4).

**Table 4:** GSEA enrichment analysis

ID	NES	pvalue	p.adjust	qvalues
REACTOME_NEUTROPHIL_DEGRANULATION	1.909562	0.001148	0.039119	0.028251
REACTOME_GPCR_LIGAND_BINDING	1.98536	0.001155	0.039119	0.028251
REACTOME_SIGNALING_BY_INTERLEUKINS	1.587387	0.001156	0.039119	0.028251
REACTOME_G_ALPHA_I_SIGNALLING_EVENTS	1.742828	0.001168	0.039119	0.028251
REACTOME_NEURONAL_SYSTEM	2.001512	0.001188	0.039119	0.028251
REACTOME_CLASS_A_1_RHODOPSIN_LIKE_RECEPTORS_	1.958376	0.001208	0.039119	0.028251
NABA_SECRETED_FACTORS	2.126348	0.001209	0.039119	0.028251
WP_NUCLEAR_RECEPTORS_METAPATHWAY	1.900179	0.00122	0.039119	0.028251

### Co-expression Modules of DEGs Identified by WGCNA

**Fig. 5:**
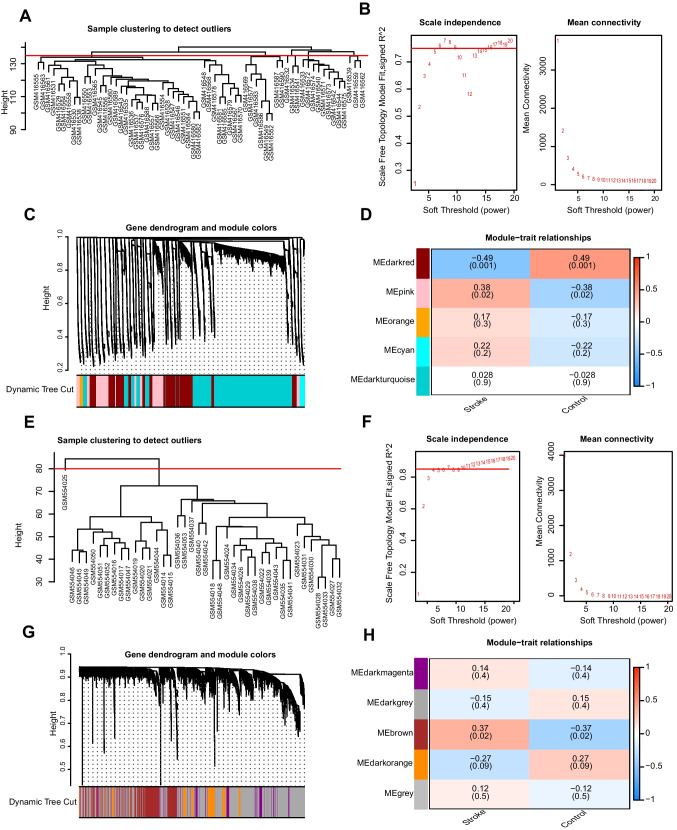
WGCNA identifies co-expression modules related to cerebral infarction. **a** No outlier specimens were found in the GSE16561 dataset by cut height. **b** Selection of the optimal soft threshold of the GSE16561 dataset. **c** The aggregation process of modular genes in the GSE16561 dataset. **d** Association between modular genes and cerebral infarction in the GSE16561 dataset. **e** No outlier specimens were found in the GSE22255 dataset by cut height. **f** Selection of the optimal soft threshold of the GSE22255 dataset. **g** The aggregation process of modular genes in the GSE22255 dataset. **h** Association between modular genes and cerebral infarction in the GSE22255 dataset

We performed WGCNA on the control and AIS groups to screen for co-expression modules. In the process of analyzing the GSE16561 dataset using WGCNA, we successfully identified 23 outlier samples by setting the cut height (Fig. 5a). After employing a scatter plot, it was found that the optimal soft threshold for our study was 5. Next, we carried out further investigations based on this finding (Fig. 5b). The co-expressed genes in the two groups were subsequently clustered in the dark red and pink modules (Fig. 5c). According to the expression pattern and grouping information of module genes, we found that the dark red and pink modules were positively correlated with ischemic stroke with *p* < 0.05 and were used for further analyses (Fig. 5d). During WGCNA of the GSE22255 dataset, an outlier sample was detected by setting the cut height (Fig. 5e). Using a scatter plot, we determined that a soft threshold of 7 provided optimal results, prompting us to proceed with subsequent investigations (Fig. 5f). The genes co-expressed in the two groups were subsequently clustered in the brown and dark orange modules (Fig. 5g). According to the expression patterns and grouping information of the module genes, we found that the brown and dark orange modules were positively correlated with cerebral infarction with *p* < 0.05 and were subjected to further analyses (Fig. 5h).

### Protein–Protein Interaction Network

**Fig. 6:**
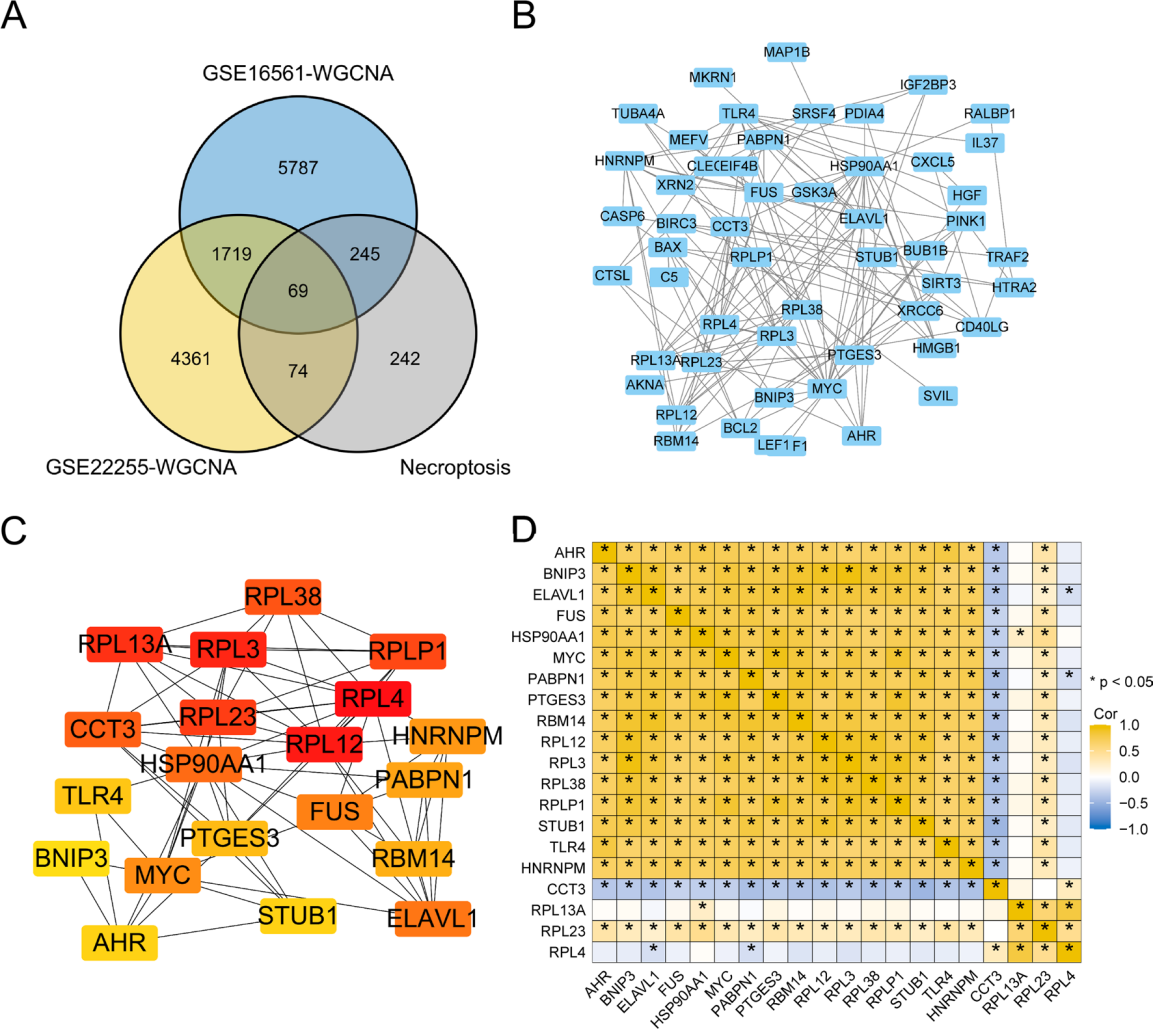
PPI network. **a** Venn diagram of the stroke-related genes of the datasets GSE16561 (blue circle) and GSE22255 (yellow circle) analyzed by WGCNA, and the necroptosis-related genes (gray circle). The genes at the intersection of the three sets of genes were designated as NRDEGs. **b** PPI network of the NRDEGs. **c** Top 20 genes screened by the CytoHubba Screening Maximum Correlation Criterion. **d** Heat map of correlation coefficient for hub genes

A total of sixty-nine genes were obtained upon the intersection of AIS-related co-expressed genes in the GSE16561 and GSE22255 datasets and necroptosis-related genes (Fig. 6a). The PPI network of AIS-related genes was constructed, and the data were plotted using Cytoscape software (Fig. 6b). Using the MCC algorithm of cytoHubba, a Cytoscape plugin, the top 20 hub gene scores were calculated. These top 20 genes included *RPL4*, *PTGES3*, *RBM14*, *HSP90AA1*, *RPLP1*, *HNRNPM*, *RPL38*, *CCT3*, *PABPN1*, *RPL13A*, *ELAVL1*, *RPL12*, *FUS*, *AHR*, *RPL23*, *STUB1*, *TLR4*, *RPL3*, *BNIP3*, and *MYC* (Fig. 6c). We generated a correlation coefficient heatmap to visualize the relationships among the hub genes (Fig. 6d).

### Construction of a Stroke Diagnostic Model and Determination of Eigengenes

**Fig. 7:**
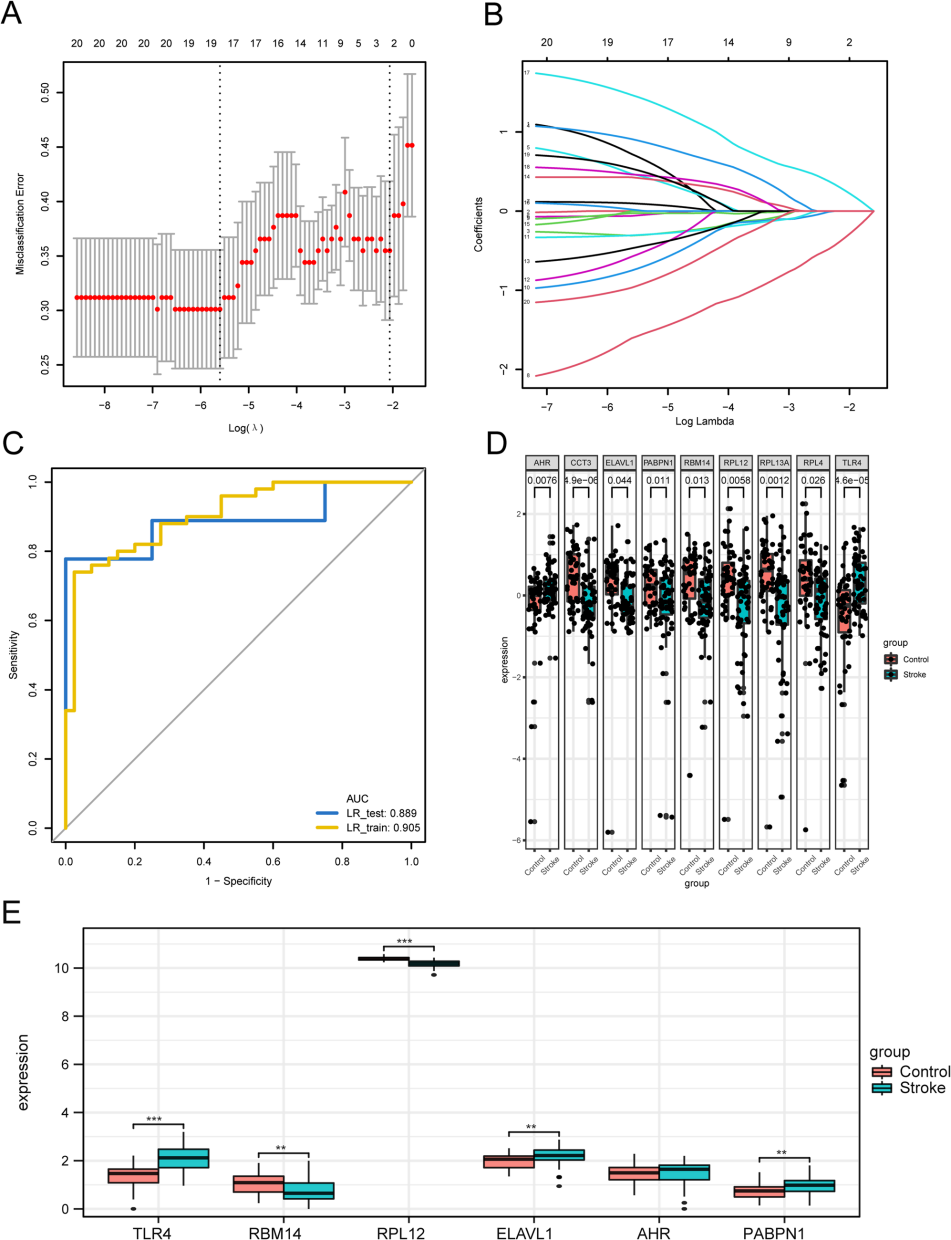
Establishment and validation of LASSO regression diagnostic model. **a** Obtaining the optimal and parsimonious models through LASSO regression. **b** Association between the selected features and the absolute value of the coefficient. **c** Model validation using both the training and test groups. **d** The characteristic genes of the cerebral infarction and control groups were significantly different. **e** The results of differential expression were displayed by group comparison plots

In order to identify AIS-associated genes and assess their diagnostic potential, we employed the LASSO regression model on the GSE_combine dataset. The dataset was randomly divided into Training and Test groups in a 7:1 ratio. The model was constructed using the training group and validated using the test group. Throughout the model-building process, the chosen feature parameters reduced as λ increased, whereas the absolute values of the coefficients increased (Fig. 7a and b). Through the process of simulation and careful selection, we identified a set of 18 feature genes, namely, *RPL4*, *RBM14*, *HSP90AA1*, *RPLP1*, *HNRNPM*, *RPL38*, *CCT3*, *PABPN1*, *RPL13A*, *ELAVL1*, *RPL12*, *FUS*, *AHR*, *STUB1*, *TLR4*, *RPL3*, *BNIP3*, and *MYC*. ROC curves were plotted for both datasets and the area under the curve (AUC) was computed to validate the model. The AUC values of the risk model for both training and test groups were 0.905 and 0.889, respectively (Fig. 7c). We analyzed the differences between the characteristic genes of the AIS group and control groups and identified nine genes with statistically significant differences (*p* < 0.05), including *RPL4*, *RBM14*, *CCT3*, *PABPN1*, *RPL13A*, *ELAVL1*, *RPL12*, *AHR*, and *TLR4*. Boxplots are shown in Fig. 7d. Next, the expression differences between the two groups were verified by dataset GSE58294. We used the Wilcoxon rank sum test to analyze the expression differences of the six hub genes (*TLR4*, *RBM14*, *RPL12*, *ELAVL1*, *AHR*, *PABPN1*). The results of expression differences were shown by the group comparison graph (Fig. 7e). The results showed that the expression levels of *TLR4*, *RBM14*, and *RPL12* were significantly different and the expression trends remained consistent in both test and validation datasets.

### Two Distinct Modes of Necroptosis Identified by Signature Genes

**Fig. 8:**
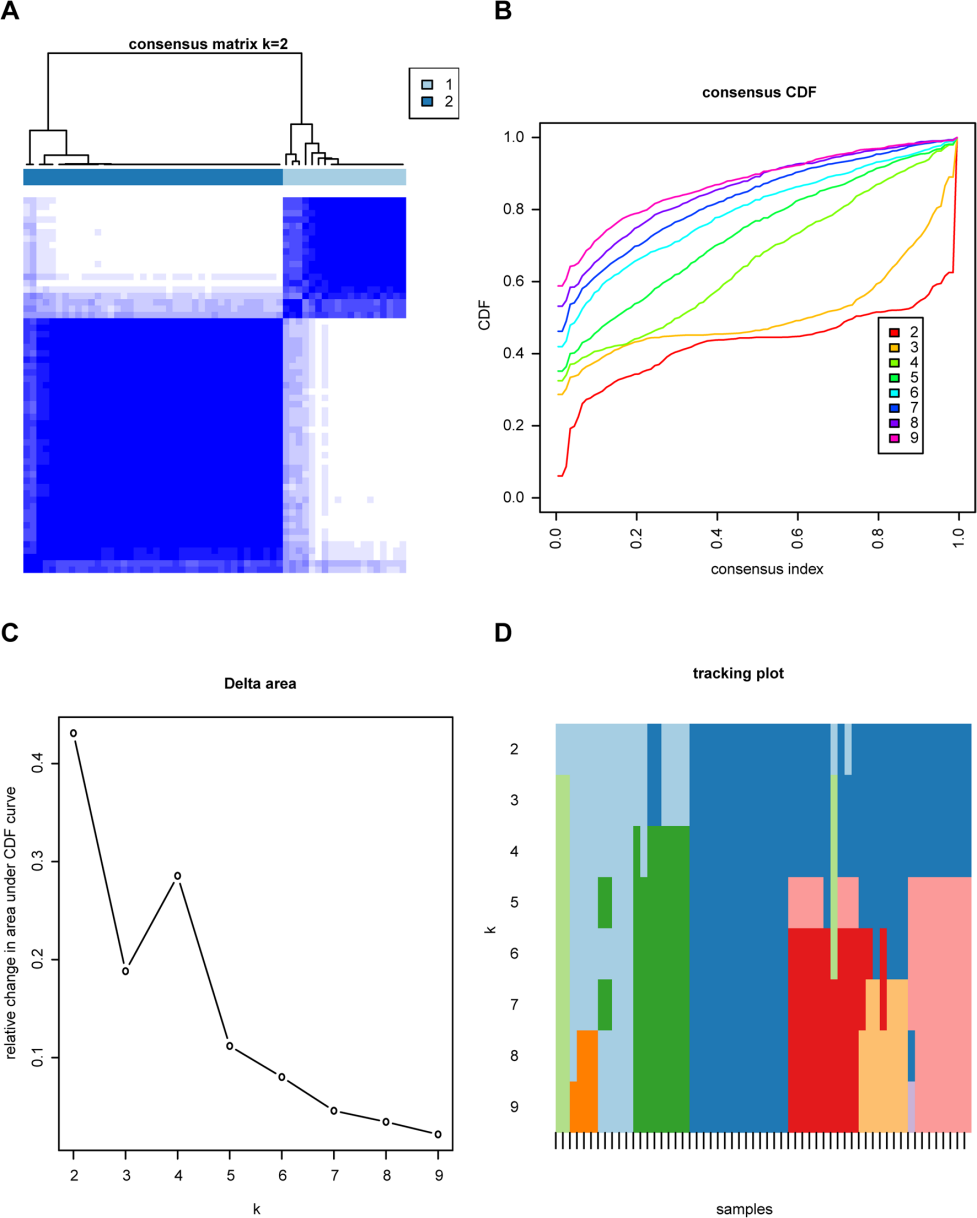
Consensus cluster analysis of eigengenes in patients with cerebral infarction. **a** Consensus clustering diagram (*k* = 2). **b** Relative variation of area under the CDF curve for *k* = 2–9. **c** Consensus clustering CDF. **d** Tracking plot

Consensus clustering of the nine necroptosis-related genes was carried out, and a consensus clustering diagram was drawn when *k* = 2 (Fig. 8a). Furthermore, we observed variations in the area under the cumulative distribution function (CDF) curve in relation to *k* = 2–9 (Fig. 8b). Additionally, we plotted the CDF of the consistent clustering (Fig. 8c) and the tracking plot (Fig. 8d).

### Immune Infiltration Analysis

**Fig. 9:**
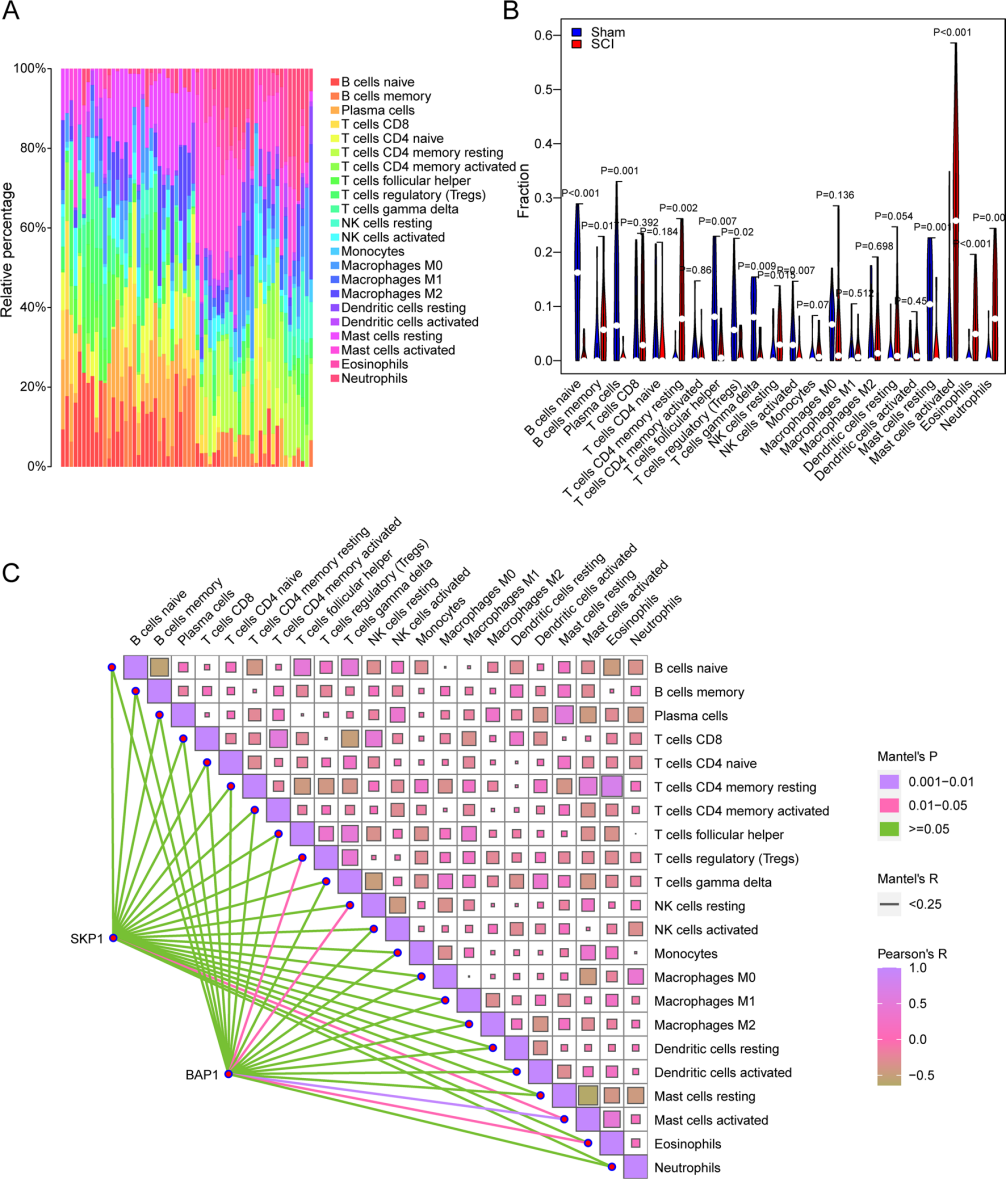
Immune infiltration analysis. **a**, **b** Variations in the abundance of 22 immune cell infiltrates across samples in the GSE_combine dataset. **b** Variations in the abundance of 22 immune cell infiltrates in the GSE_combine dataset. Blue, control group; red, patients with cerebral infarction. **c** Heatmap showing the correlations among the 22 immune cell infiltrates as well as the correlations between the 22 cell infiltrates and hub-mRNA

To analyze disparities in the extent of immune infiltration of immune infiltration between patients with AIS and the control group, variations in the abundance of 22 immune cell infiltrates between the two groups were explored using the CIBERSORT algorithm in the GSE_combine dataset (Fig. 9a). Through the use of the Wilcoxon signed-rank test algorithm, a total of nine types of immune cells in the GSE_combine dataset were found to be significantly different between patients with AIS and the Control group (Fig. 9b): plasma cell, regulatory T cell (Treg), CD8 + T cell, activated NK cell, γδ T cell, M0 macrophage, M2 macrophage, neutrophil, and activated mast cell. Next, the correlations between the nine NRDEGs and 22 types of immune cells were analyzed. In the GSE_combine dataset, significant correlations were found between *RPL4* and activated CD4 + memory T cell; *RBM14* and activated dendritic cell; *CCT3* and naïve B cell and activated mast cell; *RPL13A* and plasma cell, Treg, M1 macrophage, activated mast cell, and activated dendritic cell; *AHR* and naïve CD4 + T cell and monocyte; and *TLR4* and monocyte and M2 macrophage (Fig. 9c).

### Network for the Interactions Among Hub-mRNA, Hub-miRNA, and Hub-lncRNA

**Fig. 10:**
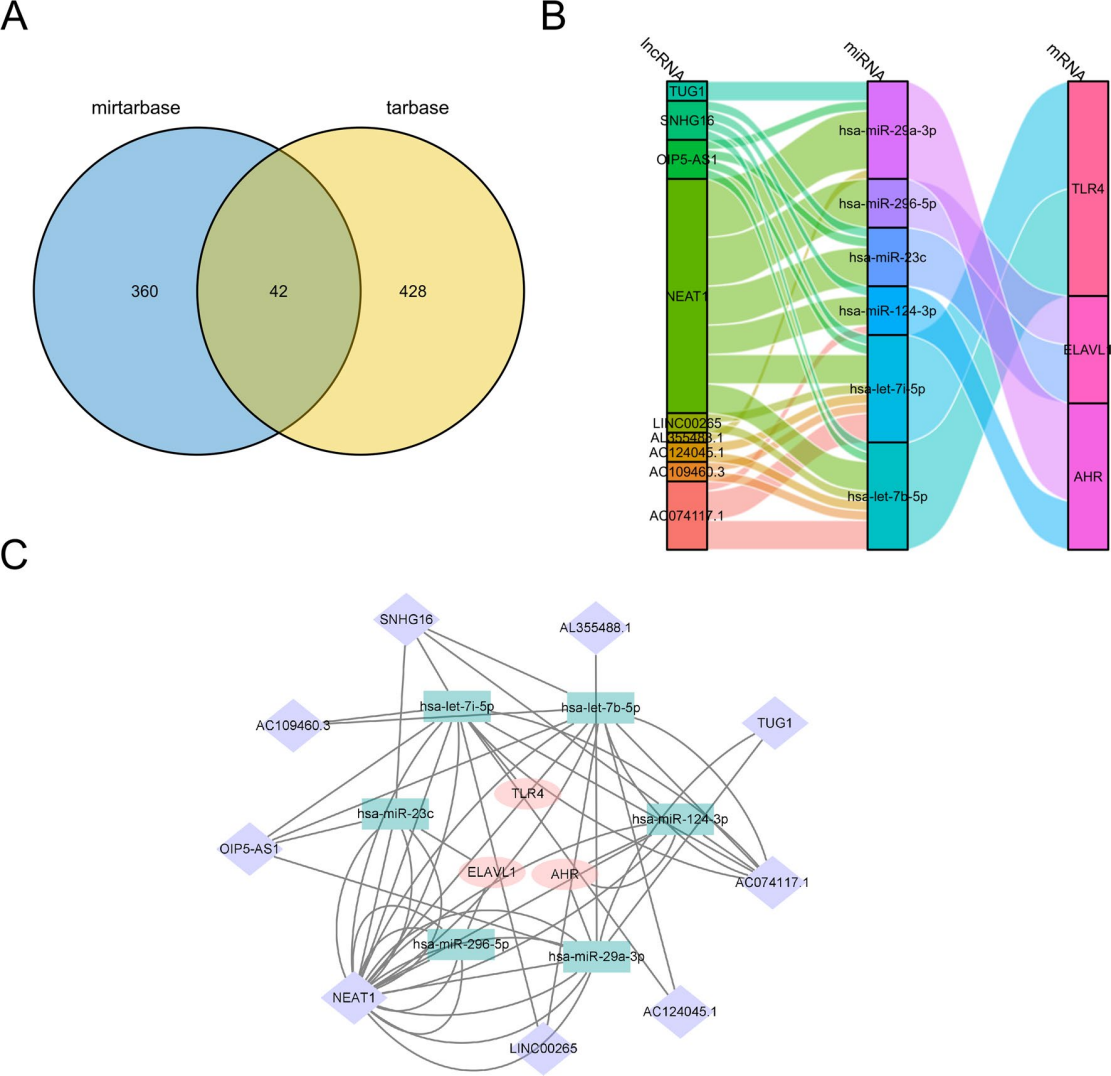
Network for the interactions among hub-mRNA, hub-miRNA, and hub-lncRNA. **a** Venn diagram shows the miRNAs predicted to interact with hub-mRNAs according to the miRTarBase and TarBase databases. There are 42 groups of interactions in the intersection. **b**, **c** lncRNAs interacting with miRNAs were predicted using the starBase database. **b** Sankey and **c** network diagrams of the lncRNA-miRNA-mRNA network were drawn

We constructed an lncRNA-miRNA-mRNA interaction network, which contained three genes related to necroptosis, namely, *TLR4, AHR*, and *ELAVL1*. We then employed the miRTarBase and TarBase databases to predict miRNAs that could interact with the necroptosis-related genes. A total of 42 sets of interactions were obtained from the intersection set (Fig. 10a), and seven sets of interactions supported by experimental evidence were identified. The lncRNAs that potentially bind to miRNAs were predicted using the starBase database, and the lncRNA-miRNA-mRNA network was established using both Sankey (Fig. 10b) and network (Fig. 10c) diagrams.

### Validation of the Identified mRNAs

**Fig. 11:**
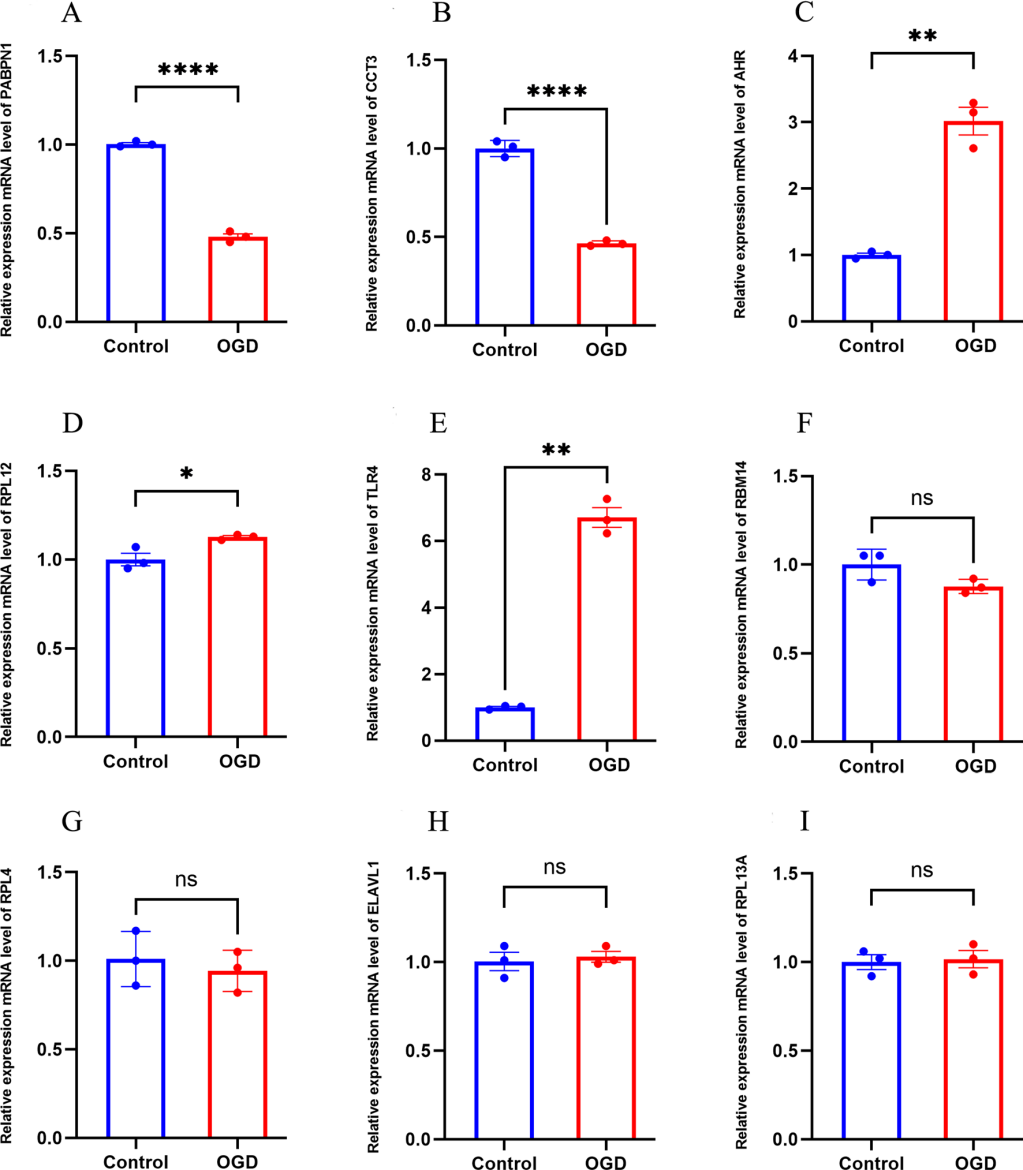
The relative expression of differentially expressed mRNA in HT22. **a** PABPN1; **b** CCT3; **c** AHR; **d** RPL12; **e** TLR4; **f** RBM14; **g** RBL4; **h** ELAVL1; **i** RPL13A. The control group reflects the normal HT22 and the OGE/R group reflects the model group. *p < 0.05, **p < 0.01, ***p < 0.001

**Fig. 12:**
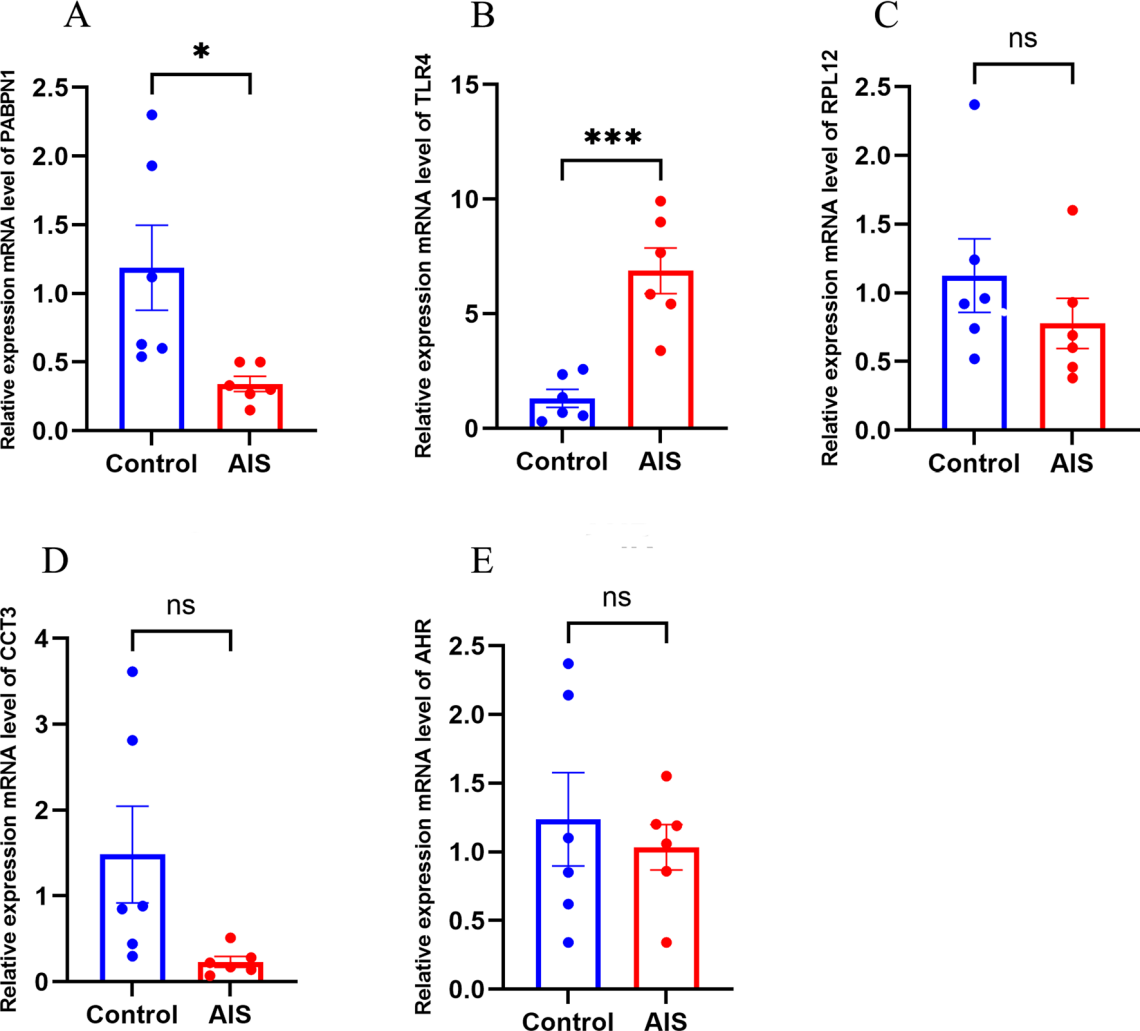
Relative expression of differentially expressed mRNA in AIS patients and healthy controls. **a** PABPN1; **b** TLR4; **c** RPL12; **d** CCT3; **e** AHR。 The control group reflected normal people, and the AIS group reflected patients with acute ischemic stroke. *p < 0.05, **p < 0.01, ***p < 0.001

Oxygen–glucose deprivation/re-oxygenation (OGD/R) was used to mimic neural injury. When compared with the normal control group, the mRNA expression levels of PAPBN1 (*p* < 0.01), CCT3 (*p* < 0.01) in the model group were significantly downregulated while the mRNA expression levels of AHR (*p* < 0.01), RPL12 (*p* < 0.05), and TLR4 (*p* < 0.01) were significantly upregulate (Fig. 11). To further verify the expression of the identified genes, we collected whole blood samples from patients with AIS and healthy controls, extracted RNA, and performed qPCR. Compared with the normal control group, the mRNA expression level of PAPBN1 (*p* < 0.05) was significantly downregulated, while the mRNA expression level of TLR4 (*p* < 0.001) was significantly upregulated in the AIS group (Fig. 12).

## Discussion

AIS is one of the main causes of human death and is one of the most prevalent leading to disability worldwide. The death of neurons can occur through various pathways, including apoptosis, necroptosis, autophagy, ferroptosis, and pyroptosis. Necroptosis is believed to be a type of necrotic cell death that occurs after cerebral infarction and I/R injury. Several studies have shown that necroptosis occurs within one hour after reperfusion in a brain injury model of I/R, primarily affecting neurons in the hippocampus (Li et al. 2020c; Naito et al. 2020). Moreover, necroptosis is associated with the size of the infarct and the extent of neurological impairment (Nikseresht et al. 2019; Han et al. 2019). The inhibition of RIPK1, genetically or pharmacologically, can alleviate ischemic brain injury (Naito et al. 2020), and a specific inhibitor, known as Nec-1, can target necroptosis. Hence, to identify effective biomarkers for diagnosing and treating AIS through necroptosis, 1945 DEGs were retrieved from the combined dataset of GSE16561 and GSE22255. From GeneCards, 630 genes related to necroptosis were obtained, and 76 genes were found to be both DEGs and NRDEGs. GO, KEGG, and GSEA were utilized to enrich the DEGs in the TNF pathway, TLR pathway, and other pathways associated with necroptosis. However, relying solely on differentially expressed genes does not necessarily capture the full range of interrelationships and regulatory networks between genes. Therefore, we further used the WGCNA method, which aims to gain a deeper understanding of the regulatory mechanisms of genes by identifying gene co-expression modules. In Fig. 4 and subsequent analysis, we performed WGCNA analysis on the GSE16561 and GSE22255 datasets, respectively, and identified co-expressed gene modules that were significantly associated with cerebral infarction. We believe that the genes in these modules may have important biological functions through their co-expression patterns. We then further intersected these co-expressed gene modules with necroptosis-related genes to produce 69 genes. PPI analysis resulted in the identification of 20 hub genes. Using LASSO regression, a diagnostic model for STROKE was constructed, determining 18 characteristic genes. Through consensus clustering, two distinct patterns of necroptosis were identified based on the signature genes. However, the precise mechanism underlying the role of necroptosis in ischemic stroke remains unclear, necessitating further research. Although RIPK1, RIPK3, and MLKL were not directly analyzed, we can indirectly infer that the hub genes may be associated with the necroptotic pathways by systematically analyzing the genes and protein networks associated with acute ischemic infarction. Some hub genes in our PPI network are involved in signaling pathways or molecular processes related to RIPK1, RIPK3, and MLKL, and regulate the expression and activity of these key proteins, such as FAS (CD95) that is involved in the activation of RIPK1, CASP8 (Caspase-8) that may also be involved in necroptosis under the regulation of RIPK1 and RIPK3, and TNFRSF10A/B that may be involved in the regulation of RIPK1. In addition, we found that differentially expressed genes were mainly related to biological processes such as immune response and cell death, which may be related to the regulation of the activity of RIPK1, RIPK3, and MLKL. Therefore, our aim was to systematically study NRDEGs, providing a better framework for exploring diagnostic biomarkers and making predictions regarding targeted therapy associated with necroptosis for the treatment of AIS.

NRDEGs were found to be enriched in the GO CC term of the cytoplasmic portion and the myosin complex, which aligns with the characteristics of necroptosis, leading to the rupture of the plasma membrane (Cai et al. 2014; Nganga et al. 2019). Additionally, the NRDEGs showed enrichment in the GO MF terms microfilament motor activity and death receptor activity. During the progression of necroptosis, the assembly of complex “necrosomes” within cells can be triggered by death receptors. Furthermore, KEGG analysis of NRDEGs demonstrated that TNF signal transduction was the main enriched pathway. TNF plays a pivotal role in the cellular events during necroptosis (Chen and Goeddel 2002), and the mouse fibroblast L-M cell line is highly susceptible to TNF-induced necroptosis (Laster et al. 1988). GSEA analysis also indicated significant enrichment of interleukin signaling, neutrophil degranulation, and G alpha (i) signaling among the NRDEGs. It has been suggested that inflammation triggered by necroptosis may be driven by inflammasome activation signaling mediated by interleukins (ILs). For instance, in an in vivo experiment, necroptosis-induced IL-1 was found to contribute to necroptosis-driven inflammation in dendritic cells due to the deletion of the key RIPK3-MLKL necroptosis suppressor caspase-8 (Kang et al. 2013). Neutrophils, which are involved in neural plasticity, have been observed to increase after a stroke (Amulic et al. 2012). TNF-induced neutrophil necroptosis has been reported to depend on RIPK1-RIPK3-MLKL signaling (Wicki et al. 2016). Neutrophil degranulation may also activate the fibrinolytic system in patients with ischemic stroke (Wicki et al. 2016). Additionally, necroptosis can be triggered by TNF (Liu et al. 2014). A study demonstrated that G alpha (i) could inhibit the effect of TNF. The enrichment of G alpha (i) signaling suggests its potential involvement in the necroptosis process (Earl et al. 1990). Our research results, revealed for the first time, the involvement of G alpha (i) signaling in the process of necroptosis in AIS. Nevertheless, further studies are needed to fully understand the underlying mechanism.

Herein, we applied the WGCNA method to construct five co-expression modules for the two datasets. Each module consisted of a set of genes exhibiting similar expression patterns. To assess the correlation between these modules and AIS, we examined the expression profiles and grouping information of the module genes. Notably, the dark red and pink modules, as well as the brown and dark orange modules, demonstrated the strongest correlations. Subsequently, we employed the ischemic stroke-related genes within these modules and intersected them with the necroptosis-related genes. The overlapping genes obtained from this intersection were subjected to further analysis.

LASSO regression models were utilized to screen eigengenes and calculate AUC values under the ROC curve for constructing a diagnostic model of ischemic stroke. The aim was to explore the predictive performance of the risk models. Using the 18 NRDEGs selected through LASSO regression analysis, we successfully constructed a diagnostic model for ischemic stroke, which exhibited promising potential for diagnostic prediction. The model’s performance was evaluated by plotting ROC curves and calculating the AUC values, which resulted in an AUC value of 0.905 for the Training group and 0.889 for the test group. Importantly, this predictive model based on the 18 NRDEGs displayed excellent predictive power, with an AUC value of 0.905 in the training group, surpassing the significance observed in previous studies. The risk model comprised nine statistically significant NRDEGs with diagnostic capabilities, namely, *RPL4*, *RBM14*, *CCT3*, *PABPN1*, *RPL13A*, *ELAVL1*, *RPL12*, *AHR*, and *TLR4*. After external dataset validation and PCR validation, only one gene TLR4 was statistically significant. Although only one gene has been shown to be significantly different, this may also indicate the importance of this gene in the biological process and deserve further study and exploration. It is well known that the protein encoded by this gene is a member of the Toll-like receptor (TLR) family, which plays a fundamental role in innate immune activation.

Functional enrichment analyses performed on the NRDEGs revealed an enrichment of GO BP terms related to the TRIF-dependent and MyD88-independent TLR pathways. Previous research suggests that necroptosis can be induced through TLR4 or TLR3 pathways. TLR3 specifically relies on TRIF for its activation, participating in NF-κB activation and induction of type I IFN (Takeuchi and Akira 2010). TLR4 is transmitted through TRIF signals and MyD88 adaptor (Takeuchi and Akira 2010). Our analysis results highlighted TLR4 as a hub gene, with GO BP terms primarily enriched in both TRIF-dependent and MyD88-independent TLR pathways. Consistent clustering is a method of exploring the underlying structure of a sample and discovering the natural clustering that exists in the data. According to Fig. 8A, at *k* = 2, the consistency matrix diagram shows that the samples are divided into two subpopulations (or two patterns) with significantly different patterns of agreement, indicating that they may be substantially different at the level of gene expression. Cluster analysis provides an initial pattern recognition to help researchers understand the underlying structure and organization of data. The expression of the TLR4 gene showed gene expression differences in two subpopulations divided by consensus cluster analysis, and one of the patterns may play a role in innate immune modulation in necroptosis of acute ischemic stroke and may be a potential therapeutic target. This provides further evidence that TLR4 activation may induce these signaling pathways, which play a role in the occurrence and progression of necroptosis. These findings align with our research results, supporting the significant involvement of NRDEGs in the progression of AIS.

The immune infiltration analysis revealed notable changes in the infiltration levels of various immune cell types in the cerebral infarction group. Specifically, activated NK cell, γδ T cell, M0 macrophage, M2 macrophage, neutrophil, and activated mast cell exhibited increased infiltration levels, while plasma cell, CD8 + T cell, and Treg showed decreased infiltration levels. It is known that neutrophils and monocytes/macrophages infiltrate and accumulate in the microvasculature and ischemic brain parenchyma (Garcia et al. 1994). γδ T cells, which are a significant population of lymphocytes, reside on the epithelial surface and possess innate immune characteristics. They secrete IL-17 and generate chemotaxis signals that attract peripheral bone marrow cells, such as neutrophils and monocytes, exacerbating ischemic brain injury (Shichita et al. 2009; Gelderblom et al. 2012). Treg cells have been shown to exert neuroprotective effects by suppressing inflammation following ischemic brain injury (Liesz et al. 2015), while CD8 + T cells tend to accumulate in the brain after a stroke (Ahnstedt et al. 2020). In addition, Selvaraj et al. demonstrated that CD8 + T cells contribute to functional recovery in the chronic phase following a stroke (Selvaraj et al. 2021). Interestingly, our findings indicate that genes such as *RPL4*, *RBM14*, *CCT3*, *RPL13A*, *AHR*, and *TLR4* are closely associated with the abundance of immune cell infiltration. This suggests their potential involvement in immune responses related to ischemic stroke.

Non-coding RNAs (ncRNAs), especially lncRNAs and miRNAs, serve as crucial regulators of gene expression. Previous studies have highlighted the involvement of specific ncRNAs in promoting inflammation and modulating the prognosis of AIS. For instance, *TUG1* was found to promote inflammation by competitively binding to miR-145, resulting in the upregulation of AQP4 expression in OGD/R cell models and McAO rat models (Du et al. 2021). Another study suggested that the lncRNA *NEAT1* may act as a sponge for miR-124 and miR-125a, negatively modulating inflammation and influencing the prognosis of AIS (Li et al. 2020a). In our study, we established a potential ceRNA regulatory network involving three NRDEGs (*TLR4*, *ELAVL1*, *AHR*), providing new insights into AIS diagnosis and more effective targeted therapies against these NRDEGs. Manipulating the expression of these necroptosis-related genes, such as *TLR4*, using miRNAs or lncRNA could potentially aid in reducing ischemia–reperfusion injury or the extent of cerebral infarction.

Although our necroptosis-related genes exhibited promise as accurate diagnostic and therapeutic targets, there are still certain limitations that need to be addressed. Firstly, subgroup analyses should include the clinical and demographic characteristics of more patients with AIS to validate the generalizability and clinical significance of these patterns. Secondly, the sample size is relatively small, and only external datasets and in vitro experiments are used for validation, which may lead to one-sided results and a high false positive rate. Thirdly, given that the relationship between necroptosis and cerebral infarction remains unclear, additional research is needed to elucidate the potential mechanisms involving necroptosis-related genes in the context of cerebral infarction.

## Conclusions

In summary, our study identified a set of novel necroptosis-associated gene signatures that hold promise for accurate diagnosis and treatment of AIS. These gene signatures not only shed light on the underlying biological changes occurring in AIS but also have the potential to serve as valuable indicators for clinical decision-making. Among the identified genes, *RPL4*, *RBM14*, *CCT3*, *PABPN1*, *RPL13A*, *ELAVL1*, *RPL12*, *AHR*, and *TLR4* emerged as key players in necroptosis-related processes. These genes hold considerable potential as biomarkers for diagnosing and treating AIS.

## Supplementary Information

Below is the link to the electronic supplementary material.

Supplementary file1 (ZIP 1708 KB)

## Data Availability

No datasets were generated or analysed during the current study.
