# Supplementary material for: Deciphering Necroptosis-Associated Molecular Subtypes in Acute Ischemic Stroke Through Bioinformatics and Machine Learning Analysis
Source: J Mol Neurosci. 2025 Jan 2;75(1):4. doi: 10.1007/s12031-024-02241-3 (PMC11693621; doi:10.1007/s12031-024-02241-3)
Supplement: Supplementary file 1 — Supplementary file1 (ZIP 1708 KB) [file 12031_2024_2241_MOESM1_ESM.zip › Supplemental Files/primer sequence.docx]

| **引物名称** | **序列** |
| --- | --- |
| **Mus-ACTIN-F** | **CTTTGCAGCTCCTTCGTTGC** |
| **Mus-ACTIN-R** | **CCTTCTGACCCATTCCCACC** |
| **Mus-RPL4-F1** | **TTACAAGAAGACCAAGGAGGCT** |
| **Mus-RPL4-R1** | **ATACCAGGGATGTTGCGGA** |
| **Mus-RBM14-F2** | **CCAAATCCCCGCTTACCAC** |
| **Mus-RBM14-R2** | **AGCACCCTTCCACACCCAC** |
| **Mus-CCT3-F2** | **TCCAGGATGCCATGCAAGTG** |
| **Mus-CCT3-R2** | **CGAAGGGAGGTAAGCAGACGA** |
| **Mus-RPL13A-F1** | **GTTTGCTTACCTGGGGCGTCT** |
| **Mus-RPL13A-R1** | **ACATTCTTTTCTGCCTGTTTCCG** |
| **Mus-ELAVL1-F2** | **CTCAAGGGGTCAGGAATCG** |
| **Mus-ELAVL1-R2** | **AGGTGGAAGTGGGAGGGTC** |
| **Mus-RPL12-F1** | **ATCGGTCCTCTGGGTCTGTC** |
| **Mus-RPL12-R1** | **TTCTGCTTCTTCCTGTCTCTCG** |
| **Mus-AHR-F1** | **CCAGCCCTTTCTCTCCCATAAT** |
| **Mus-AHR-R1** | **ACTCCCGCACTTGCTCACG** |
| **Mus-TLR4-F** | **CACTGTTCTTCTCCTGCCTGAC** |
| **Mus-TLR4-R** | **GACTTTGCTGAGTTTCTGATCCAT** |
| **Mus-PABPN1-F** | **TGTGGTTCAGTCAACCGTGT** |
| **Mus-PABPN1-R** | **CCATGATGTCGCTCTAGCCC** |

| **引物名称** | **序列** |
| --- | --- |
| **Human-ACTIN-F** | **GCACCGTCAAGGCTGAGAAC** |
| **Human-ACTIN-R** | **TGGTGAAGACGCCAGTGGA** |
| **Human-RPL12:F** | **AGG CAA CGG GTG ACT GGA AG** |
| **Human-RPL12:R** | **TGT TTC TTT CTG TCT CTT GGT GGT TC** |
| **Human-PABPN1:F** | **CGA GGT AGA GAA GCA GAT GAA TAT GAG** |
| **Human-PABPN1:R** | **ACC ATA GTC CAC ATT GCC AAC ATA G** |
| **Human-CCT3:F** | **ATT GTG CTG CTG GAT TCT TCT CTG** |
| **Human-CCT3:R** | **TGA AGT CCT CCT CTC GTG TAA TCT C** |
| **Human-AHR:F** | **GCT CTG AAT GGC TTT GTA TTA GTT GTC** |
| **Human-AHR:R** | **CTC GGT CTT CGG TAT GGA TAA GTT C** |
| **Human-TLR4:F** | **TCT TGG TGG AAG TTG AAC GAA TGG** |
| **Human-TLR4:R** | **AGC ACA CTG AGG ACC GAC AC** |
