# Supplementary figures and images for: Deciphering Necroptosis-Associated Molecular Subtypes in Acute Ischemic Stroke Through Bioinformatics and Machine Learning Analysis
Source: J Mol Neurosci. 2025 Jan 2;75(1):4. doi: 10.1007/s12031-024-02241-3 (PMC11693621; doi:10.1007/s12031-024-02241-3)

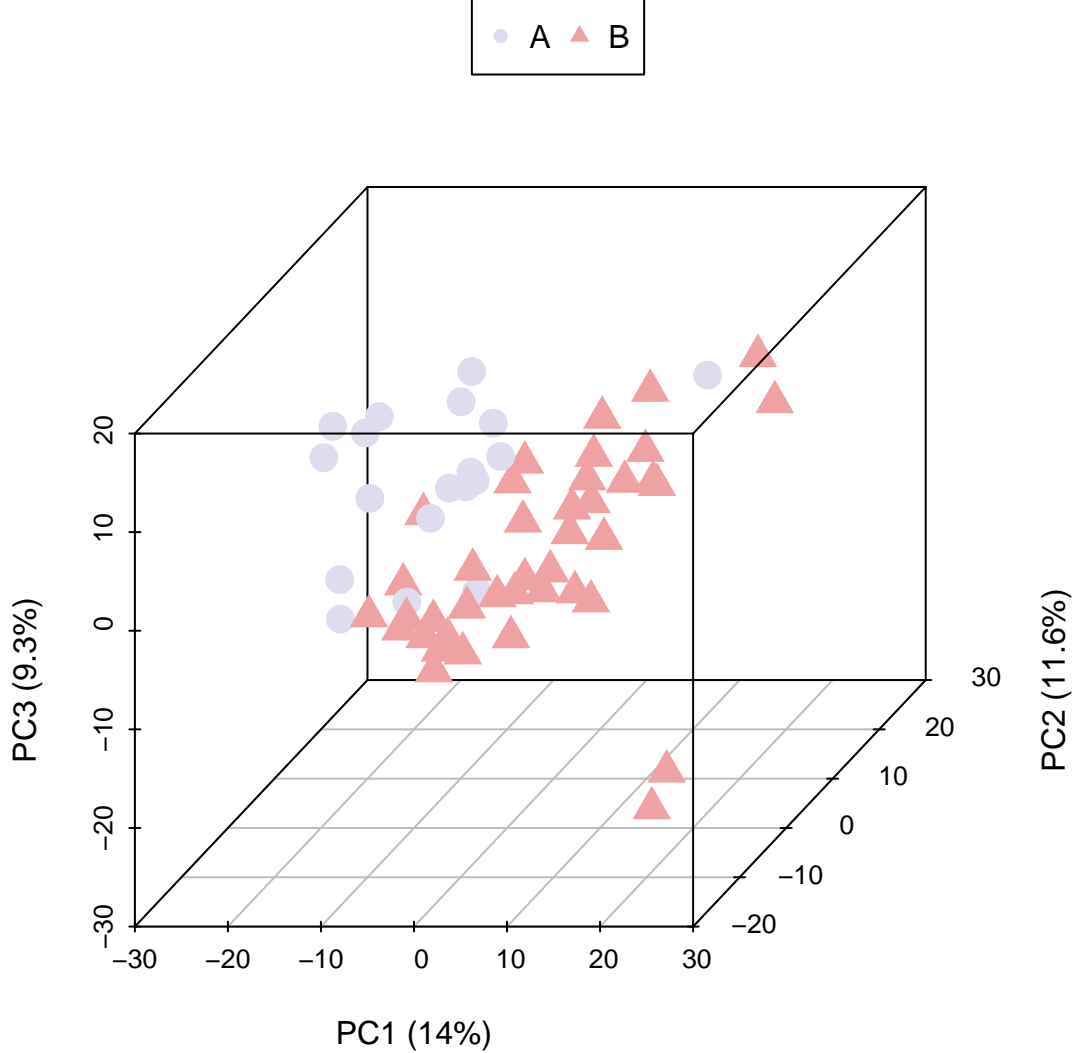

Supplement: Supplementary file 1 — Supplementary file1 (ZIP 1708 KB) [file 12031_2024_2241_MOESM1_ESM.zip › Supplemental Files/1-3D-PCA.pdf]

Before

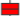 GSE16561    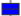 GSE22255

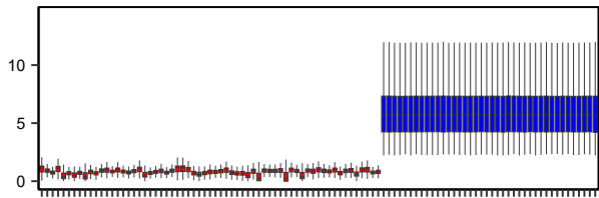

Supplement: Supplementary file 1 — Supplementary file1 (ZIP 1708 KB) [file 12031_2024_2241_MOESM1_ESM.zip › Supplemental Files/Boxplot_Before.pdf]

After

GSE16561 GSE22255

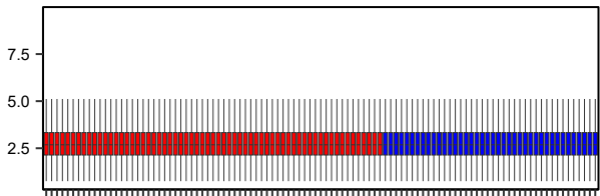

Supplement: Supplementary file 1 — Supplementary file1 (ZIP 1708 KB) [file 12031_2024_2241_MOESM1_ESM.zip › Supplemental Files/Boxplot_after.pdf]

# GSE16561

● Up ● Not sig ● Down

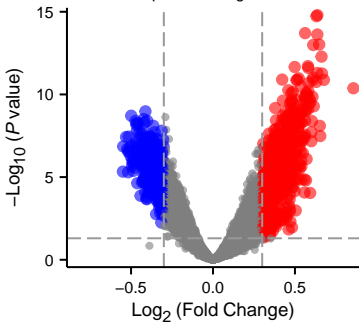

Supplement: Supplementary file 1 — Supplementary file1 (ZIP 1708 KB) [file 12031_2024_2241_MOESM1_ESM.zip › Supplemental Files/GSE16561火山图.pdf]

# GSE22255

● Up    ● Not sig    ● Down

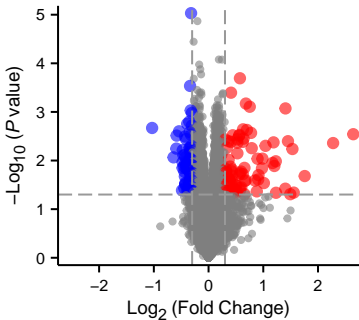

Supplement: Supplementary file 1 — Supplementary file1 (ZIP 1708 KB) [file 12031_2024_2241_MOESM1_ESM.zip › Supplemental Files/GSE22255火山图.pdf]

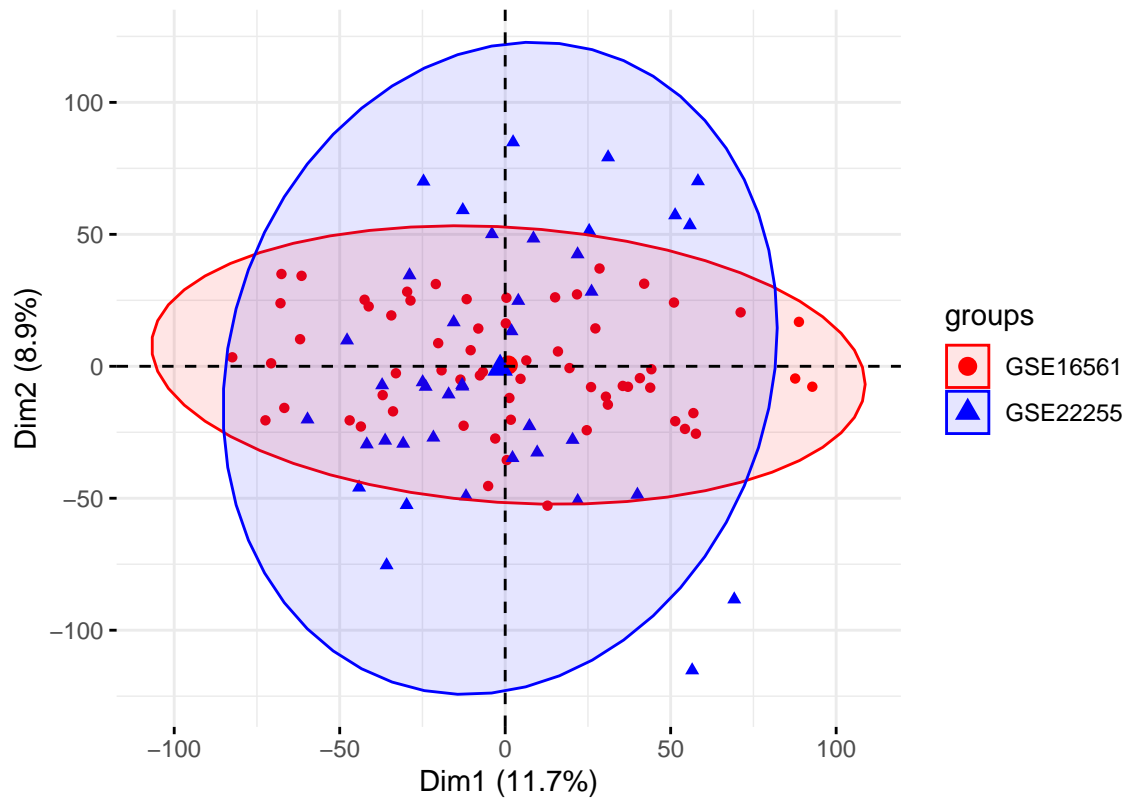

Supplement: Supplementary file 1 — Supplementary file1 (ZIP 1708 KB) [file 12031_2024_2241_MOESM1_ESM.zip › Supplemental Files/PCA_After.pdf]

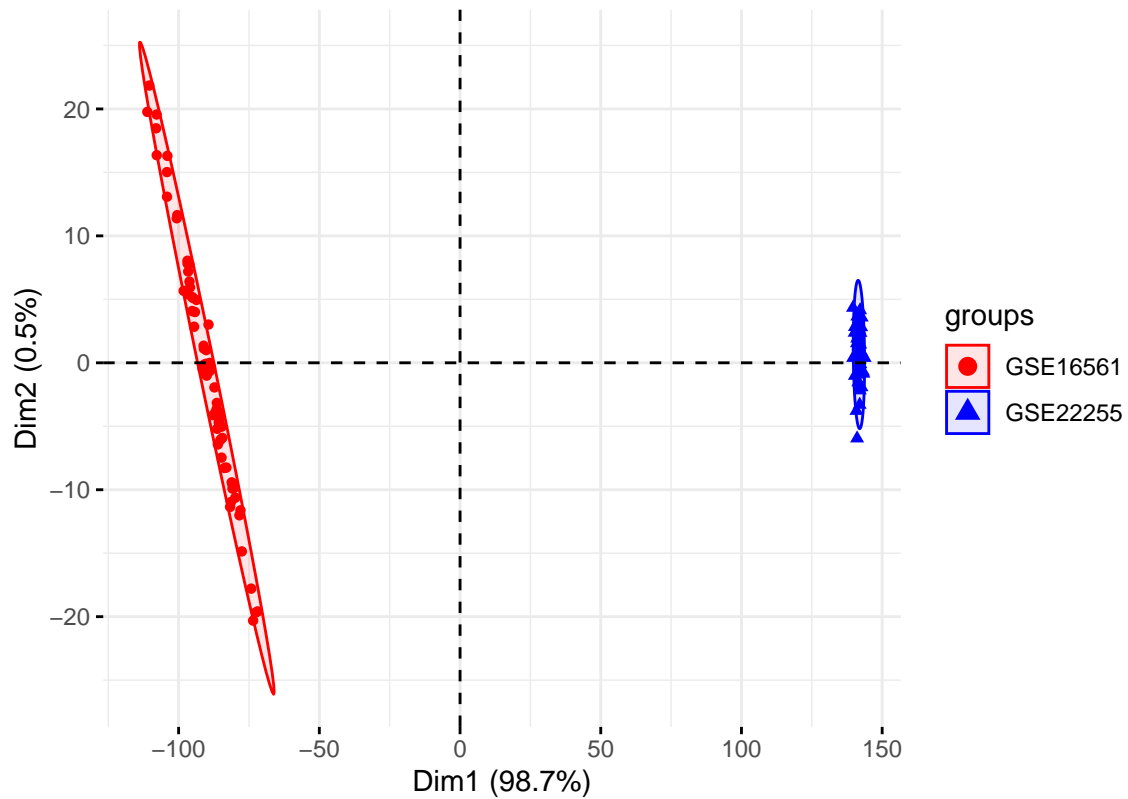

Supplement: Supplementary file 1 — Supplementary file1 (ZIP 1708 KB) [file 12031_2024_2241_MOESM1_ESM.zip › Supplemental Files/PCA_Before.pdf]
